# Change in Prolactin Levels in Pediatric Patients Given Antipsychotics for Schizophrenia and Schizophrenia Spectrum Disorders: A Network Meta-Analysis

**DOI:** 10.1155/2018/1543034

**Published:** 2018-04-01

**Authors:** Chakrapani Balijepalli, Eric Druyts, Michael J. Zoratti, Ping Wu, Salmaan Kanji, Kiran Rabheru, Kevin Yan, Kristian Thorlund

**Affiliations:** ^1^Faculty of Health Sciences, Simon Fraser University, Burnaby, BC, Canada; ^2^Faculty of Medicine, University of British Columbia, Vancouver, BC, Canada; ^3^Department of Clinical Epidemiology and Biostatistics, McMaster University, Hamilton, ON, Canada; ^4^Canadian College of Naturopathic Medicine, Toronto, ON, Canada; ^5^Department of Pharmacy, The Ottawa Hospital and Ottawa Hospital Research Institute, Ottawa, ON, Canada; ^6^Department of Psychiatry, Ottawa Hospital, Ottawa, ON, Canada

## Abstract

**Background:**

Treatment of schizophrenia with first- and second-generation antipsychotics has been associated with elevated prolactin levels, which may increase the risk for prolactin-related adverse events.

**Methods:**

Randomized controlled trials (RCTs) included in a recent systematic review were considered for this analysis. A Bayesian network meta-analysis was used to compare changes in prolactin levels in pediatric patients diagnosed with schizophrenia or schizophrenia spectrum disorders treated with second-generation antipsychotics (SGAs).

**Results:**

Five RCTs, including 989 patients combined, have evaluated the changes in prolactin for pediatric patients after 6 weeks of treatment with risperidone, quetiapine, aripiprazole, olanzapine, and paliperidone. In the overall study population, treatment with risperidone was associated with the highest increase in mean prolactin levels compared to other SGAs. Patients treated with risperidone 4–6 mg/day were found to experience the greatest increases (55.06 ng/ml [95% CrI: 40.53–69.58]) in prolactin levels, followed by risperidone 1–3 mg/day, paliperidone 3–6 mg/day, and paliperidone 6–12 mg/day.

**Conclusions:**

This study shows that there are differences in SGAs ability to cause hyperprolactinemia. Further, there is clear evidence of safety concerns with risperidone and paliperidone treatment in adolescent schizophrenia patients.

**Registration:**

PROSPERO CRD42014009506.

## 1. Background

Schizophrenia has a lifetime prevalence of 0.3% to 0.7% and an incidence of 10.2 to 22 per 100,000 person-years with varying rates depending on the diagnostic criteria used [1]. Schizophrenia is considered to be a rare disorder in childhood, but it becomes increasingly common in adolescence. Early-onset schizophrenia that occurs in children and adolescents can be divided into very-early-onset schizophrenia (occurring in children aged 12 or less) and adolescent-onset schizophrenia (occurring between the ages of 13–19) [2, 3]. Prior studies have shown that only 0.1% to 1% of schizophrenic disorders start before the age of 10, 4% start before the age of 15, and nearly 10% start between the ages of 16 and 20. In patients under 15 years of age diagnosed with schizophrenia, males are affected disproportionately, at a ratio of approximately 3 : 1 [2].

Antipsychotics that block dopamine 2 receptors (D2Rs) have been the mainstay of treatment in patients diagnosed with schizophrenia. While first- and second-generation antipsychotics were proven to be quite effective in improving the psychotic symptoms, they are associated with adverse events such as neurological effects, weight gain, and increased triglycerides and cholesterol [1]. Another important safety concern reported in patients treated with antipsychotics is elevated serum prolactin levels (hyperprolactinemia). Normal serum prolactin levels are <25 ng/ml in women and <20 ng/ml in men; hyperprolactinemia is defined as fasting serum levels above the normal values [4–6]. Hyperprolactinemia is present in up to 70% of the patients receiving antipsychotic medications [7]. Although both first- and second-generation antipsychotics can cause hyperprolactinemia, the risk differs between medications, with a higher risk reported in treatments involving amisulpride, sulpiride, and risperidone [8]. Although antipsychotics are approved for adolescent schizophrenia patients, in some countries, they are prescribed off-label to children with schizophrenia without the approval of regulatory agencies [9, 10]. Problems concerning off-label prescription of antipsychotics in children have been highlighted in previous research [11, 12]. In children, hyperprolactinemia can lead to a plethora of unwanted symptoms relating to reproductive dysfunction, sexual impairment, breast pathology, hypogonadism, and behavioral and mood alterations [13].

Although previous research has shown that antipsychotics can cause a rise in prolactin levels, there is a lack of evidence regarding head-to-head comparisons of adverse event profiles of several antipsychotics in the treatment of schizophrenia in pediatric populations. Therefore, we conducted a network meta-analysis (NMA) to study the relative changes in serum prolactin levels in pediatric patients diagnosed with schizophrenia and schizophrenia spectrum disorders treated with SGAs.

## 2. Methods

We performed an NMA of randomized controlled trials (RCTs) included in a recent systematic literature review on changes in serum prolactin levels and prolactin-related adverse events in pediatric patients (PROSPERO CRD42014009506) [14]. Briefly, MEDLINE, Embase, CENTRAL, and PsycINFO databases were searched for RCTs and observational studies reporting changes in prolactin levels and prolactin-related adverse events in pediatric patients (aged 5 to 18 years) diagnosed with schizophrenia or schizophrenia spectrum disorders and treated with antipsychotics.

The findings of the systematic literature review conducted by Druyts et al. indicate that reporting of the incidence of adverse events in pediatric schizophrenia patients is limited and prolactin elevation as an adverse event is reported more often in the pediatric schizophrenia population who are on antipsychotics. Therefore, this analysis focuses on changes in prolactin levels in patients treated with SGAs based on RCT evidence. Where the change from baseline (CFB) of prolactin was not reported in the publication, it was calculated using the measures at baseline and endpoint.

An NMA was conducted in the Bayesian framework, using the conventional model setup with noninformative priors recommended by the National Institute for Health and Clinical Excellence [15]. This approach makes use of both direct and indirect evidence and allows for comparisons between treatments where no head-to-head evidence exists [16–18]. Results are presented for the fixed effect models as standardized mean CFB (95% credible interval [CrI]). For convenience and ease of reading, we took the liberty of referring to effect estimates as “statistically significant” where the 95% credible intervals preclude the null effect (although this is not strictly in line with conventional Bayesian inferences). Model parameters were estimated using Markov Chain Monte Carlo simulation in the OpenBUGS software package (http://www.openbugs.net).

## 3. Results

**Figure 1:**
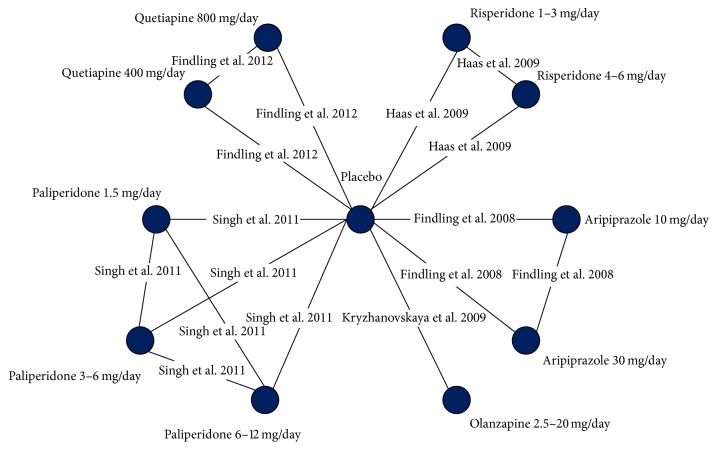
Complete evidence network of the trials included in the analyses that reported change in prolactin levels.

**Figure 2:**
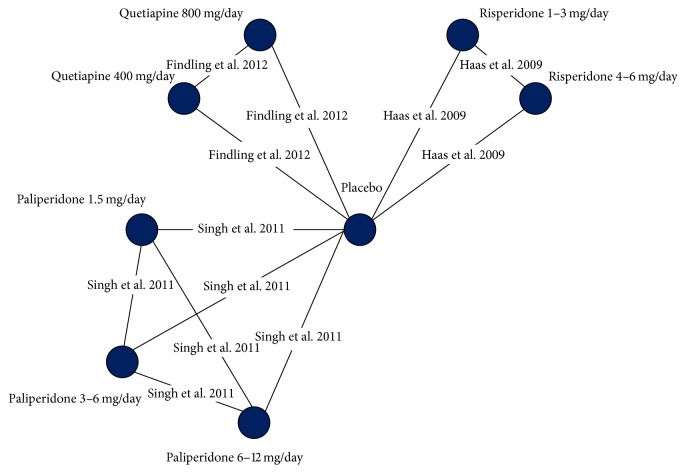
The evidence network of the trials that reported change in prolactin levels by sex.

The previously conducted systematic literature review identified an evidence base comprising 6 RCTs assessing adolescents treated with SGAs [19–24]. For the purpose of the NMA, 5 RCTs were included [19–23]. One study was excluded as it did not include a comparator arm that could be incorporated into the network of evidence [24]. All trials reported outcomes after 6 weeks of treatment. The network for the analysis of the overall population is presented in Figure 1 and the network for the analysis with results stratified by sex is presented in Figure 2.

A summary of the included studies is presented in Table 1. Baseline characteristics, presented in Table 2, were generally well balanced both between treatment arms and across trials. Patients included reported similar mean ages and Positive and Negative Syndrome Scale (PANSS) scores. All studies included a higher proportion of males and Caucasians. Patients included in the study of Kryzhanovskaya et al. (2009) had the lowest mean serum prolactin levels at baseline [22].

### 3.1. Overall Population

The results of the NMA for the overall population (*N* = 989) are presented in Table 3. The interventions included in the overall analysis include risperidone (1–3 mg/day, *n* = 55; 4–6 mg/day, *n* = 51), quetiapine (400 mg/day, *n* = 73; 800 mg/day, *n* = 74), aripiprazole (10 mg/day, *n* = 100; 30 mg/day, *n* = 102), olanzapine (2.5–20 mg/day, *n* = 72), paliperidone (1.5 mg/day, *n* = 54; 3–6 mg/day, *n* = 48; 6–12 mg/day, *n* = 47), and placebo (*n* = 313).

As presented in Table 3, when compared to placebo, all treatments, except for aripiprazole and the lower dose of paliperidone (1.5 mg/day), showed a statistically meaningful increase in prolactin. Risperidone 4–6 mg/day showed the largest increase in mean prolactin levels when compared to placebo (55.06 ng/ml [95% CrI: 40.53–69.58]), followed by risperidone 1–3 mg/day (31.28 ng/ml [95% CrI: 20.21–42.38]), paliperidone 3–6 mg/day (19.89 ng/ml [95% CrI: 9.45–30.41]), paliperidone 6–12 mg/day (19.68 ng/ml [95% CrI: 8.70–30.58]), olanzapine 2.5–20 mg/day (12.09 ng/ml [95% CrI: 5.71–18.55]), quetiapine 800 mg/day (10.40 ng/ml [95% CrI: 1.38–19.19]), and quetiapine 400 mg/day (7.68 ng/ml [95% CrI: 0.08–15.20]). Both doses of aripiprazole showed a reduction in prolactin levels when compared to placebo, although results were not statistically meaningful. Risperidone exhibited a dose effect for the change in prolactin levels, with a mean difference of 23.8 ng/ml (95% CrI: 8.31–39.37).

### 3.2. Analysis of Males Only

Only three of the included studies reported the CFB in prolactin levels by sex [19, 21, 23]. The NMA of mean CFB in prolactin levels in males included risperidone (1–3 mg/day, *n* = 30; 4–6 mg/day, *n* = 37), quetiapine (400 mg/day, *n* = 43; 800 mg/day, *n* = 44), paliperidone (1.5 mg/day, *n* = 30; 3–6 mg/day, *n* = 31; 6–12 mg/day, *n* = 33), and placebo (*n* = 185).

The results of the NMA for the studies reporting change in prolactin levels in males are presented in Table 4. Of the results that were statistically significant, risperidone 4–6 mg/day showed the highest increase in prolactin levels when compared to placebo (29.46 ng/ml [95% CrI: 17.20–41.73]), followed by paliperidone 3–6 mg/day (22.18 ng/ml [95% CrI: 10.88–33.43]), paliperidone 6–12 mg/day (20.61 ng/ml [95% CrI: 9.40–32.00]), and risperidone 1–3 mg/day (19.07 ng/ml [95% CrI: 7.38–30.93]). Additionally, paliperidone 1.5 mg/day and quetiapine 800 mg/day also exhibited a statistically nonsignificant increase in prolactin levels when compared to placebo.

### 3.3. Analysis of Females Only

Only three of the included studies reported the CFB in prolactin levels by sex [19, 21, 23]. The NMA of mean CFB in prolactin levels in females included risperidone (1–3 mg/day, *n* = 25; 4–6 mg/day, *n* = 14), quetiapine (400 mg/day, *n* = 30; 800 mg/day, *n* = 30), paliperidone (1.5 mg/day, *n* = 24; 3–6 mg/day, *n* = 17; 6–12 mg/day, *n* = 14), and placebo (*n* = 128).

The results of the NMA for the studies reporting change in prolactin levels in females are presented in Table 4. Like the results observed in the overall sample, of the results that were statistically significant, risperidone 4–6 mg/day showed the greatest increase in prolactin levels when compared to placebo (83.93 ng/ml [95% CrI: 51.04–117.00]), followed by risperidone 1–3 mg/day (45.39 ng/ml [95% CrI: 26.02–64.96]), quetiapine 400 mg/day (21.06 ng/ml [95% CrI: 7.11–34.99]), and quetiapine 800 mg/day (19.41 ng/ml [95% CrI: 0.83–37.88]). It can be observed that when compared to males, females exhibited a higher increase in the mean CFB in prolactin levels with both doses of risperidone, but the credible intervals were slightly wider. Further, when compared to placebo, paliperidone 3–6 mg/day and paliperidone 6–12 mg/day exhibited a nonsignificant increase in prolactin levels in females.

## 4. Discussion

Previous studies have reported on the hyperprolactinemia potential of antipsychotics; however, currently, there is very little head-to-head comparison evidence of antipsychotics to establish the relative differences in serum prolactin levels with antipsychotic treatments in pediatric populations. In this context, NMA is a useful technique to compare the direct and indirect evidence available to assess the relative safety of SGAs in the treatment of pediatric patients diagnosed with schizophrenia and schizophrenia spectrum disorders, specifically regarding increased serum prolactin levels. Using the NMA approach, this study has demonstrated that, in the overall study population and by sex, both doses of risperidone are associated with the greatest increase in serum prolactin levels of all treatments considered. Similar trends were observed in the analysis stratified by sex; however, higher elevations with wider 95% CrIs were observed in females. Elevations in serum prolactin were also found to be statistically meaningful in females for quetiapine, whereas in males, statistically meaningful elevations in serum prolactin were noticed with paliperidone 3–6 mg/day and paliperidone 6–12 mg/day.

Prolactin elevations with antipsychotic treatments were well studied; however, the lion's share of the research has been conducted on adult patients with first episodes of psychosis. The results from studies on adults showed that risperidone showed higher elevations in prolactin levels when compared with other SGAs such as clozapine, olanzapine, quetiapine, and ziprasidone [25–27]. Previous studies have suggested that 9-hydroxyrisperidone (paliperidone), a major metabolite of risperidone, is responsible for the increase in serum prolactin levels in the patients treated with risperidone [28, 29]. Leucht et al. (2013) have assessed the comparative efficacy and tolerability of 15 antipsychotic medications in schizophrenia patients and showed that risperidone and paliperidone have exhibited similar elevations in serum prolactin levels [30]. The same study also showed that olanzapine produced greater increases in serum prolactin levels when compared to quetiapine [30]. Although the trends for the overall study population and the males-only analysis were similar to the previous studies, differences in trends and the lack of significance for the females-only analysis could be attributed to lower proportion of females in the included trials, especially in the trials with paliperidone and quetiapine treatments.

Several hypotheses have been put forward to explain the differences in the prolactin elevation properties of different antipsychotics. Antipsychotics causing higher prolactin elevations tend to dissociate slower from the D2Rs and are also associated with a lesser degree of penetration of the blood-brain barrier at therapeutic doses [8]. Risperidone, when compared with olanzapine and quetiapine, has a slower dissociation rate from the D2R and weaker blood-brain barrier penetrating ability, resulting in higher elevations of serum prolactin levels [8]. A study by Saito et al. also confirmed the significantly higher levels of prolactin in adolescents treated with risperidone when compared to olanzapine and quetiapine [31].

Several important factors should be considered for an NMA to be valid. These include the notion that trials included in the analyses are homogenous enough to be combined and populations included in the trials are also sufficiently similar with respect to the baseline values of important effect modifiers and prognostic factors that can affect outcomes. Although some heterogeneity does exist, as is expected in any meta-analysis, the authors believe that this did not compromise the validity in the interpretation of the findings.

The findings of the NMA must be interpreted with caution as the evidence base is comprised of few studies. Further, all comparisons were only informed by a single trial. The analysis was limited to the use of an indirect outcome, namely, the change in prolactin level in treated patients, as the available evidence was not conducive to an analysis directly on adverse events.

In light of these limitations, this analysis used the best available evidence to quantify the relationship between antipsychotic medication use and changes in prolactin levels in pediatric patients. In conclusion, this study shows that there are important differences between SGAs and their ability to cause hyperprolactinemia. Further, there is further evidence of safety concerns with risperidone and paliperidone in the pediatric population. We believe that the selection of treatment for schizophrenia and schizophrenia-related disorders should incorporate a quantifiable risk, and thus monitoring of prolactin-related adverse events is pertinent. We recommend future studies of SGAs in pediatric patients to consistently report adverse events, particularly those related to prolactin levels.

## 5. Conclusions

This study shows that there are differences in SGAs' ability to cause hyperprolactinemia. Further, there is clear evidence of safety concerns with risperidone and paliperidone in adolescent patients. Studies of SGAs in adolescent patients reporting adverse events, particularly those related to prolactin levels, are warranted.

## Figures and Tables

**Table 1:** Summary of the characteristics of the included trials.

Study	Diagnosis	Criteria used for diagnosis	Treatment duration (weeks)	Follow-up assessment of prolactin levels (weeks)	Inclusion criteria
Findling et al. 2012 [19]	Schizophrenia	DSM-IV-TR + K-SADS-PL	6	6	Inpatients or outpatients aged 13–17 years; PANSS ≥ 60
Findling et al. 2008 [20]	Schizophrenia	DSM-IV	6	6	Aged 13–17 years; PANSS ≥ 70
Haas et al. 2009 [21]	Schizophrenia	DSM-IV + K-SADS-PL	6	6	Aged 13–17 years; PANSS 60–120
Kryzhanovskaya et al. 2009 [22]	Schizophrenia	DSM-IV-TR	6	6	Aged 13–17 years; BPRS ≥ 35
Singh et al. 2011 [23]	Schizophrenia	DSM-IV + K-SADS-PL	6	6	Aged 12–17 years; weight ≥ 29 kg; PANSS 60–120

DSM: Diagnostic and Statistical Manual; TR: text revision; K-SADS-PL: Kiddie-Sads-Present and Lifetime; PANSS: Positive and Negative Syndrome Scale [14].

**Table 2:** Baseline characteristics and outcome data among participants included in the trials.

Study	*N* (ITT)	Treatment	Age, mean (SD)	Male, *n* (%)	Caucasian, *n* (%)	Previous antipsychotic use, *n* (%)	Baseline PANSS score, mean (SD)	Baseline prolactin, both sexes (ng/mL), mean (SD)	Baseline prolactin, males (ng/mL), mean (SD)	Baseline prolactin, females (ng/mL), mean (SD)	Prolactin change at 6 weeks, both sexes (ng/mL), mean (SD)	Prolactin change at 6 weeks, males (ng/mL), mean (SD)	Prolactin change at 6 weeks, females (ng/mL), mean (SD)
Findling et al. 2012 [19]	73	Quetiapine 400 mg/day	15.5 (1.3)	43 (58.9)	45 (61.6)	NR	96.2 (17.7)	20.8 (17.0)	NR	NR	−10.6 (16.1)	−9.22 (14.4)	−12.4 (18.5)
	74	Quetiapine 800 mg/day	15.5 (1.3)	44 (59.5)	44 (59.5)	NR	97.0 (15.3)	18.1 (20.1)	NR	NR	−7.8 (26.5)	−3.7 (11.6)	−14.0 (39.1)
	73	Placebo	15.3 (1.4)	42 (57.5)	46 (63.0)	NR	96.7 (18.0)	28.7 (29.1)	NR	NR	−18.3 (28.8)	−6.53 (15.1)	−33.9 (34.9)

Findling et al. 2008 [20]	100	Aripiprazole 10 mg/day	15.6 (1.3)	45 (45.0)	54 (54.0)	53 (53.0)	93.7 (15.7)	NR	NR	NR	−11.9 (23.3)	NR	NR
	102	Aripiprazole 30 mg/day	15.4 (1.4)	65 (63.7)	62 (60.8)	47 (46.1)	94.9 (15.5)	NR	NR	NR	−15.1 (26.9)	NR	NR
	100	Placebo	15.4 (1.4)	61 (61.0)	64 (64.0)	46 (46.0)	95.0 (15.5)	NR	NR	NR	−8.5 (24.2)	NR	NR

Haas et al. 2009 [21]	55	Risperidone 1–3 mg/day	15.7 (1.3)	30 (54.5)	33 (60.0)	NR	NR	29.6 (38.2)	21.6 (15.2)	39.2 (53.0)	25.5 (33.5)	16 (23.7)	36.9 (41.3)
	51	Risperidone 4–6 mg/day	15.7 (1.3)	37 (72.5)	24 (47.1)	NR	NR	24.1 (23.0)	22.7 (19.9)	27.9 (28.3)	49.5 (46.9)	26.4 (28.5)	77.3 (60.8)
	54	Placebo	15.5 (1.4)	35 (64.8)	27 (50.0)	NR	NR	22.6 (23.3)	21.5 (21.1)	24.6 (25.9)	−5.9 (24.9)	−3.2 (24.8)	−9.2 (24.1)

Kryzhanovskaya et al. 2009 [22]	72	Olanzapine 2.5–20 mg/day	16.1 (1.3)	51 (70.8)	52 (72.2)	51 (70.8)	95.3 (14.1)	15.6 (12.3)	NR	NR	8.8 (17.9)	NR	NR
	35	Placebo	16.3 (1.6)	24 (68.6)	25 (71.4)	30 (85.7)	95.5 (14.1)	18.7 (16.2)	NR	NR	−3.3 (14.8)	NR	NR

Singh et al. 2011 [23]	54	Paliperidone 1.5 mg/day	15.1 (1.5)	30 (55.6)	35 (64.8)	47 (87.0)	91.6 (12.5)	25.1 (32.8)	14.7 (14.5)	38.0 (45.5)	3.3 (36.0)	3.6 (19.1)	2.9 (48.6)
	48	Paliperidone 3–6 mg/day	15.3 (1.6)	31 (64.6)	34 (70.8)	44 (91.7)	90.6 (14.0)	26.6 (43.2)	16.1 (1.8)	45.67 (71.0)	22.7 (34.1)	22.8 (30.1)	22.4 (38.7)
	47	Paliperidone 6–12 mg/day	15.5 (1.6)	33 (70.2)	32 (68.1)	40 (85.1)	91.5 (13.9)	20.8 (22.3)	18.2 (13.7)	26.88 (33.9)	22.4 (35.5)	21.3 (31.1)	24.9 (42.0)
	51	Placebo	15.7 (1.4)	23 (45.1)	35 (68.6)	48 (94.1)	90.6 (12.1)	24.5 (30.5)	13.6 (13.6)	33.43 (38.5)	2.7 (15.2)	0.6 (9.4)	4.4 (18.3)

NR: not reported; SD: standard deviation; ITT: intention to treat; PANSS: Positive and Negative Syndrome Scale [14].

**Table 3:** Network meta-analysis results of mean change in prolactin from baseline in the overall study population.

**Placebo**										
**31.28** **(20.21, 42.38)**	**Risperidone** **1–3 mg/day**									
**55.06** **(40.53, 69.68)**	**23.84** **(8.31, 39.37)**	**Risperidone** **4–6 mg/day**								
**7.68** **(0.08, 15.20)**	−**23.66** **(**−**37.14, **−**10.04)**	−**47.41** **(**−**64.00, **−**30.85)**	**Quetiapine** **400 mg/day**							
**10.40** **(1.38, 19.19)**	−**20.92** **(**−**35.20, **−**6.58)**	−**44.74** **(**−**61.78, **−**27.61)**	2.71 (−4.37, 9.78)	**Quetiapine** **800 mg/day**						
−3.46 (−10.01, 3.08)	−**34.75** **(**−**47.72, **−**21.94)**	−**58.54** **(**−**74.59, **−**42.61)**	−**11.17** **(**−**21.14, **−**1.10)**	−**13.87** **(**−**24.90, **−**2.69)**	**Aripiprazole** **10 mg/day**					
−6.68 (−13.73, 0.45)	−**37.99** **(**−**51.14, **−**24.88)**	−**61.76** **(**−**77.95, **−**45.53)**	−**14.34** **(**−**24.65, **−**3.95)**	−**17.05** **(**−**28.36, **−**5.81)**	−3.22 (−10.13, 3.71)	**Aripiprazole** **30 mg/day**				
**12.09** **(5.71, 18.55)**	−**19.21** **(**−**32.00, **−**6.23)**	−**42.99** **(**−**58.95, **−**26.96)**	4.44 (−5.51, 14.35)	1.67 (−9.20, 12.75)	**15.53** **(6.40, 24.71)**	**18.75** **(9.24, 28.34)**	**Olanzapine** **2.5–20 mg/day**			
0.60 (−9.87, 11.15)	−**30.67** **(**−**45.99, **−**15.56)**	−**54.51** **(**−**72.43, **−**36.56)**	−7.05 (−20.00, 5.82)	−9.79 (−23.40, 4.10)	4.05 (−8.37, 16.50)	7.23 (−5.38, 20.06)	−11.46 (−23.78, 0.70)	**Paliperidone** **1.5 mg/day**		
**19.89** **(9.45, 30.41)**	−11.34 (−26.66, 3.86)	−**35.19** **(**−**53.27, **−**17.23)**	12.20 (−0.61, 25.33)	9.52 (−4.14, 23.44)	**23.33** **(11.01, 35.72)**	**26.53** **(13.94, 39.19)**	7.81 (−4.40, 20.09)	**19.30** **(5.72, 32.88)**	**Paliperidone** **3–6 mg/day**	
**19.68** **(8.70, 30.58)**	−11.65 (−27.26, 3.89)	−**35.43** **(**−**53.78, **−**17.14)**	12.03 (−1.39, 25.31)	9.30 (−4.89, 23.43)	**23.15** **(10.38, 35.87)**	**26.35** **(13.40, 39.36)**	7.56 (−5.07, 20.21)	**19.07** **(4.96, 32.98)**	−0.23 (−14.10, 13.65)	**Paliperidone** **6–12 mg/day**

The results of the fixed effects NMA are displayed. Data are presented as mean difference (95% credible interval). Values correspond to the mean difference in the row versus the column. Values in bold are those differences that are statistically important.

**Table 4:** Network meta-analysis results of mean change in prolactin from baseline by patient sex.

Females only								
Males only	**Placebo**	**45.39** **(26.02, 64.96)**	**83.93** **(51.04, 117.00)**	**21.06** **(7.11, 34.99)**	**19.41** **(0.83, 37.88)**	−1.47 (−22.03, 19.02)	17.83 (−1.72, 37.39)	20.09 (−2.59, 43.13)
	**19.07** **(7.38, 30.93)**	**Risperidone** **1–3 mg/day**	**38.41** **(3.66, 73.84)**	−**24.38** **(**−**48.30, **−**0.35)**	−26.03 (−52.87, 1.00)	−**46.86** **(**−**75.20, **−**18.82)**	−**27.58** **(**−**55.01, **−**0.15)**	−25.30 (−55.39, 4.43)
	**29.46** **(17.20, 41.73)**	10.36 (−2.01, 22.94)	**Risperidone** **4–6 mg/day**	−**62.85** **(**−**98.96, **−**27.00)**	−**64.48** **(**−**102.70, **−**26.72)**	−**85.25** **(**−**124.90, **−**46.32)**	−**66.09** **(**−**104.50, **−**27.90)**	−**63.70** **(**−**103.40, **−**24.08)**
	−2.70 (−8.97, 3.59)	−**21.83** **(**−**35.26, **−**8.46)**	−**32.17** **(**−**46.08, **−**18.40)**	**Quetiapine** **400 mg/day**	−1.69 (−17.11, 13.71)	−22.52 (−47.18, 2.14)	−3.20 (−27.30, 20.91)	−0.90 (−27.88, 25.88)
	2.81 (−2.95, 8.54)	−**16.27** **(**−**29.36, **−**3.18)**	−**26.65** **(**−**40.29, **−**13.13)**	5.54 (−0.04, 11.05)	**Quetiapine** **800 mg/day**	−20.80 (−48.43, 6.66)	−1.48 (−28.68, 25.29)	0.67 (−28.60, 29.87)
	3.04 (−4.82, 10.87)	−**16.05** **(**−**30.30, **−**2.08)**	−**26.43** **(**−**41.22, **−**11.94)**	5.71 (−4.30, 15.74)	0.23 (−9.52, 9.86)	**Paliperidone** **1.5 mg/day**	19.32 (−7.47, 45.84)	21.50 (−7.73, 50.80)
	**22.18** **(10.88, 33.43)**	3.04 (−13.14, 19.30)	−7.33 (−23.97, 9.28)	**24.87** **(11.96, 37.86)**	**19.35** **(6.66, 31.87)**	**19.16** **(6.50, 31.75)**	**Paliperidone** **3–6 mg/day**	2.32 (−25.95, 31.03)
	**20.61** **(9.40, 32.00)**	1.54 (−14.70, 17.62)	−8.87 (−25.30, 7.58)	**23.35** **(10.46, 36.24)**	**17.82** **(5.19, 30.41)**	**17.59** **(4.98, 30.30)**	−1.52 (−16.40, 13.56)	**Paliperidone** **6–12 mg/day**

The results of the fixed effects NMA are displayed. Data are presented as mean difference (95% credible interval). Values correspond to the mean differences in the column versus the row for the upper triangle and the mean differences in the row versus the column for the lower triangle. Values in bold are those differences that are statistically important.

## Data Availability

All data generated or analyzed during this study are included in this published article.
